# The effectiveness of early surgical stabilization for multiple rib fractures: a multicenter randomized controlled trial

**DOI:** 10.1186/s13019-023-02203-7

**Published:** 2023-04-10

**Authors:** Zhengwei Wang, Yifei Jia, Mi Li

**Affiliations:** 1 Department of Thoracic Surgery, The 904th Hospital of PLA Joint Logistic Support Force, Xing Yuan North Road 101, Wuxi, 214044 China; 2 Department of Thoracic Surgery, Affiliated Hospital of Jiangnan University, Wuxi, 214044 China

**Keywords:** Multiple rib fractures, Rib fracture fixation, 30-Day all-cause mortality, Postoperative complications, RCT, Outcome

## Abstract

**Introduction:**

Multiple rib fractures (≥ 3 displaced rib fractures and/or flail chest) are severe chest trauma with high morbidity and mortality. Rib fixation has become the first choice for multiple rib fracture treatment. However, the timing of surgical rib fixation is unclear.

**Materials and methods:**

The present study explored whether early rib fracture fixation can improve the outcome of multiple rib fractures. The present research included patients who were hospitalized in three Jiangsu hospitals following diagnosis with multiple rib fractures. Patients received early rib fracture fixation (≤ 48 h) or delayed rib fracture fixation (> 48 h) utilizing computer-based random sequencing (in a 1:1 ratio). The primary outcome measures included hospital length of stay, intensive care unit (ICU) stay, mechanical ventilation, inflammatory cytokine levels, infection marker levels, infection, and mortality.

**Results:**

A total of 403 individuals were classified into two groups, namely, the early group (n = 201) and the delayed group (n = 202). Patients belonging to the two groups had similar baseline clinical data, and there were no statistically significant differences between them. Early rib fracture fixation greatly decreased the length of stay in the ICU (4.63 days vs. 6.72 days, *p* < 0.001), overall hospital stay (10.15 days vs. 12.43 days, *p* < 0.001), ventilation days (3.67 days vs. 4.55 days, *p* < 0.001), and hospitalization cost (6900 USD vs. 7600 USD, *p* = 0.008). Early rib fracture fixation can decrease inflammatory cytokine levels and infection marker levels, prevent hyperinflammation and improve infection in patients with multiple rib fractures. The timing of rib fracture fixation does not influence the surgical procedure time, operative blood loss, 30-day all-cause mortality, or surgical site infection.

**Conclusion:**

The findings from the present research indicated that early rib fracture fixation (≤ 48 h) is a safe, rational, effective and economical strategy and worth clinical promotion.

## Introduction

Multiple rib fractures are severe chest traumas caused by falls, motor vehicles, and deceleration events that often lead to chest wall collapse, abnormal breathing, and other complications and even endanger the patient's life [1–3]. A rib fracture occurs in 10–15% of patients who sustain blunt trauma, and it is associated with a morbidity and mortality rate of up to 25%. Multiple rib fracture-associated complications included atelectasis, pain, pneumonia, and respiratory failure, which worsened patients’ pulmonary function and long-term recovery and prolonged the intensive care unit (ICU) stay and hospital length of stay [4–6]. Kent [7] reported that rib fractures are the most serious injury in more than half of elderly patients who died of chest injuries. Thus, appropriate clinical evaluation, management strategies, and treatment play a very important role in MRF patients.

Traditionally, multiple rib fractures are managed conservatively with mainly supportive agents, such as analgesia, mechanical ventilation, and pulmonary hygiene [8, 9]. It remains controversial whether multiple rib fractures should be surgically treated to achieve stability [10, 11], and the clinical efficacy and surgical stabilization timing are also controversial [12, 13]. Pieracci [14] reported a prospective, controlled clinical evaluation and found that surgical stabilization of multiple rib fractures can improve acute outcomes compared with the best medical management. Additionally, many recent systematic reviews and meta-analyses have found the benefit of surgical rib fixation for outcomes compared to conservative management, which is highly recommended as the first choice for multiple rib fractures [12, 13, 15, 16]. To our knowledge, the timing of surgical rib fixation is unclear. Becker [11] reported that most MRF patients presented rib fracture fixation for more than 48 h, while there may be an increased rate of complications with longer intensive care units (ICUs) and hospital times. Recently, a matched-pairs analysis of the German trauma registry showed that early rib fracture fixation can decrease ICU stay and shorten the duration of ventilator use [17]. Regrettably, almost all of the studies had small sample sizes or were retrospective studies. Hence, a larger, multicenter, controlled study with the best design is needed to clarify whether early rib fracture fixation can improve MRF patient prognosis.

Thus, we suppose that early rib fracture fixation can decrease the length of stay in the ICU and overall hospital stays, thereby decreasing the inflammatory response and protecting against infection. The present study aimed to test whether the timing of surgical rib fixation affected postoperative outcomes in a multicenter randomized controlled trial.

## Methods

### Study design

**Fig. 1:**
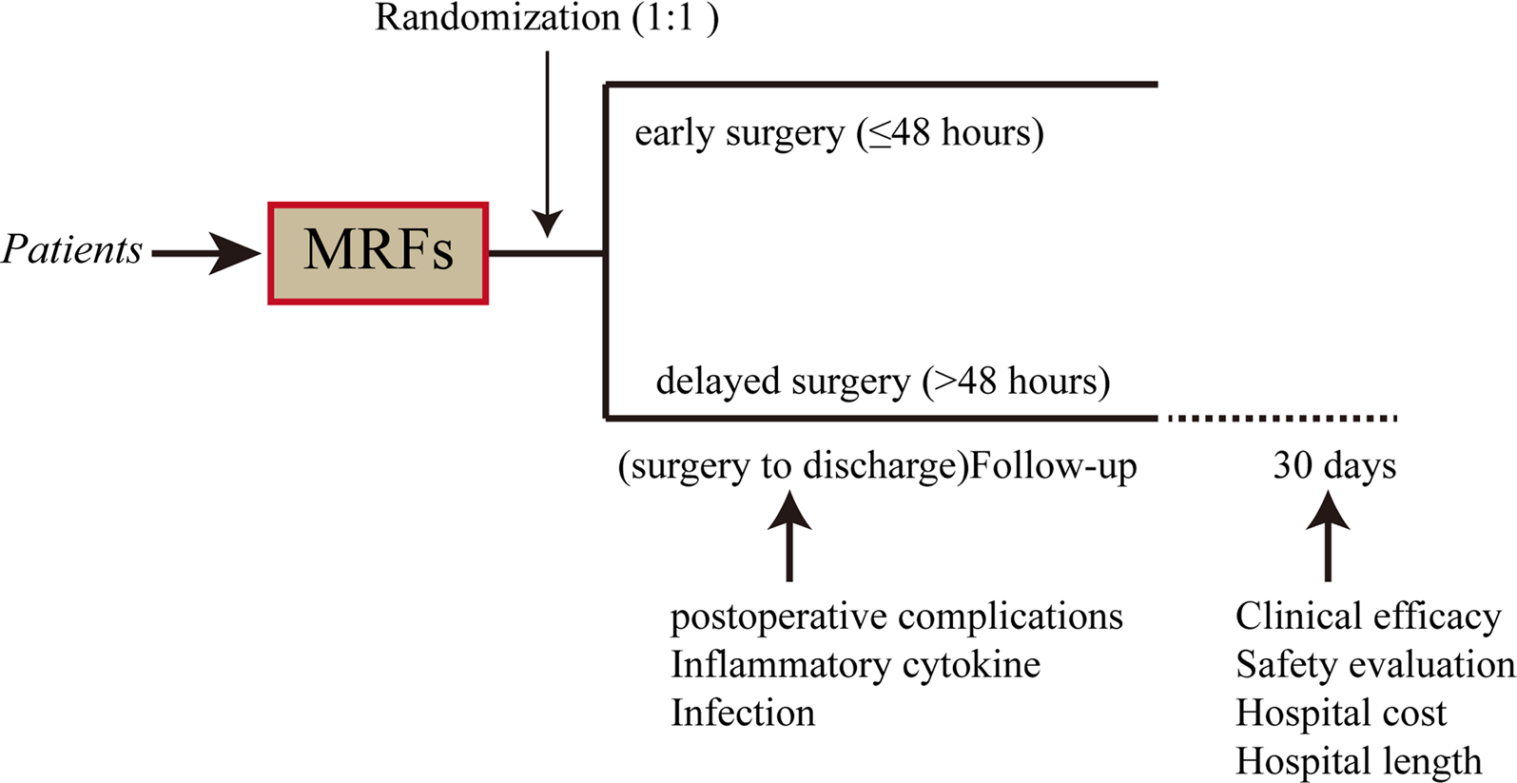
Study design

A multicenter, controlled, parallel-arm, randomized trial was carried out in Jiangsu Province from Jan 2018 to December 2021. A total of 446 patients were screened over this period, and 403 of these were initially enrolled in the study to form the ITT (intention-to-treat) population. To determine if the intervention is superior, the present research was conducted. The Clinical Research Ethics Committees of Wuxi Taihu Hospital, Huishan Peoples Hospital, and Wuxi Peoples Hospital endorsed the methodology used in the present research (2018-YXLL-072) and followed the Declaration of Helsinki. All patients were asked to obtain written informed consent for the study. In addition, patients were allocated at random (1:1) to receive early rib fracture fixation (≤ 48 h) or delayed rib fracture fixation (> 48 h, Fig. 1). All patients presented received CT three-dimensional reconstruction examination and presented plate procedures. All other treatments were the same. The last check-up was performed 30 days following the procedure.

### Patients enrolled in the study and sample selection procedures

Patients were included in the present research in three hospitals in Jiangsu Province. The following were the criteria for inclusion: (1) patients aged 18 to 70 years; (2) patients who met the diagnostic criteria of multiple rib fractures by imaging examination (CT three-dimensional reconstruction), multiple rib fractures in patients with multiple (≥ 3) displaced rib fractures and/or flail chest (≥ 3 consecutive ribs fractured in ≥ 2 places) [18]; (3) patients with complete data; and (4) patients who could be randomly assigned to receive either early rib fracture fixation or delayed rib fracture fixation. The exclusion criteria were as follows: (1) patients who were unlikely to be salvaged upon admission, such as patients who were near death or unable to be resuscitated; (2) patients with other severe traumas, such as severe traumatic brain injury, severe spinal cord injury, or cardiac injury; (3) coagulation disorders; (4) immune and inflammatory diseases before multiple rib fractures; (5) other organ failures, such as heart failure, hepatic failure, and kidney failure; (6) pregnant women and patients with malignant tumors; (7) treatment with immunosuppressive drugs; and (8) other explanations discovered by researchers, such as participation in other studies or inability to follow doctor's orders.

### Randomization and concealment

With the aid of SPSS software (version: 14.0) (SPSS Institute, Hefei, Anhui Medical University), permuted-block randomization was carried out based on a computer system that used an allotment list to produce random numbers (in a one-to-one ratio). This was carried out by a statistician who was not a member of the research team to maintain the integrity and blinding of the research. The outcomes of the random sampling process were enclosed in prenumbered envelopes and kept at the location of the research until the study’s conclusion was reached. All patients were randomized upon admission to the hospital when they were diagnosed and determined to require surgery by two senior surgeons. All of the occurrences were recorded in detail. Then, we acquired information on the patient's demographics, medical histories, and pertinent investigation findings.

### Surgical planning and fixation

There are two orthopedic trauma surgeons in three centers with a special interest in this field who perform surgical fixation of rib fractures. Before enrolling patients, all surgeons at different hospitals were professionally trained. Fractures in the third to tenth ribs were deemed suitable for fixation. One of the 3 standard surgical approaches was used for access to rib fractures chosen for fixation: lateral thoracotomy, a posterior paramedian approach, and an inframammary approach [18]. Most patients received thoracoscope surgery, and a titanium rib fracture fixation system was used. After implantation of the metal work and closure in layers, an intercostal chest drain was placed and left in situ for 24–48 h.

### Bare actual endpoints assessment

All clinical and imaging data and treatment were subjected to assessment by a masked independent diagnostic and assessment committee. This committee included two researchers who were trained before the start of the present research and did not engage in the clinical care of patients. The primary endpoints of this study were the overall duration of ICU/hospital stay and healthcare costs among patients belonging to the two groups. Additionally, we also evaluated the operation time, amount of blood loss during the operation, ICU length of stay, mechanical ventilation time, and 30-day all-cause mortality rate. The secondary endpoints included the following: (1) Inflammatory cytokine levels (pretherapy and posttreatment), including serum tumor necrosis factor-α (TNF-α) and interleukin-6 (IL-6). (2) Serum infection marker levels, such as C-reactive protein (CRP) and procalcitonin (PCT).

### Complication evaluation

We kept track of the length of time spent in the intensive care unit and general ward, and the most prevalent complications were assessed by comparing the occurrence of surgical site infection, pneumonia, and atelectasis. All complications were confirmed and recorded by physical examination by two doctors and nurses. Finally, we checked the related physical examination and imaging examination over the 30 days.

### Sample size estimates

In a previous study [19], the primary endpoint showed that the overall duration of hospital stay was 23.44 days in the early surgery group and 28.50 days in the delayed surgery group. The sample size was calculated according to an alpha of 0.05 and a statistical power of 80%, and 384 patients were enrolled (192 in each category). We decided to enroll 400 patients (200 in each category). Ultimately, we enrolled 201 patients in the early surgery group and 202 patients in the delayed surgery group. The study database included all baselines, and outcome data were entered by a study nurse.

### Statistical analysis

Data from the baseline as well as outcome assessments were input into the database by a research nurse. The information was gathered on handwritten forms and stored in a digital database that was password secured. All continuous variables are presented as the mean ± SD. SPSS 19.0 statistical software (SPSS, Inc., Chicago, USA) was used for the statistical analyses. Measurement data with a nonnormal distribution are represented by M (Q1, Q3). Independent-samples t tests were used to assess quantitative data. Qualitative data were compared with the chi-square test or Fisher’s exact t test. A value of *p* < 0.05 was considered statistically significant.

## Results

**Fig. 2:**
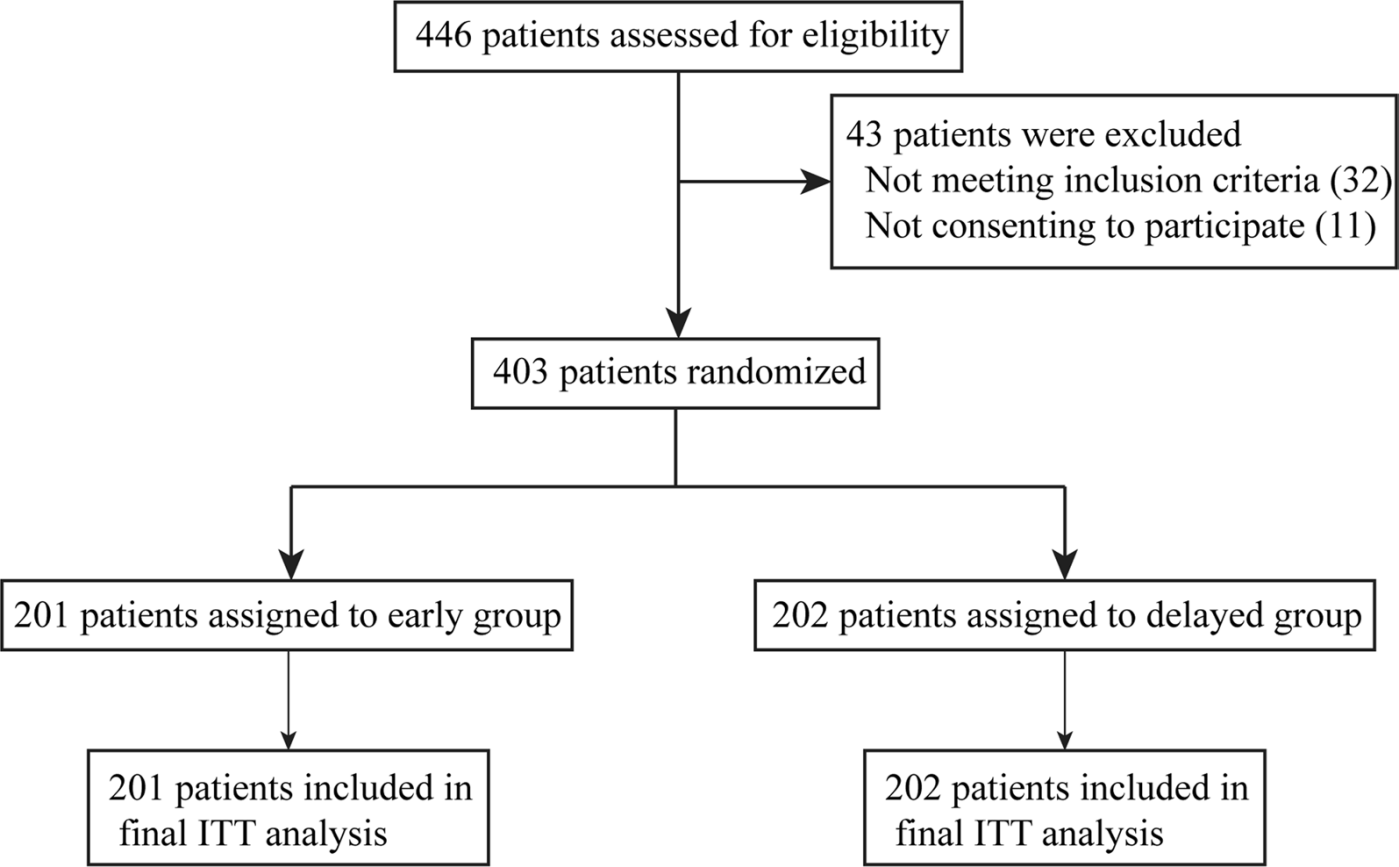
Trial profile

A total of 446 patients with multiple rib fractures were evaluated between Jan 2018 and December 2021, and all patients received CT three-dimensional reconstruction examination and the present plate procedure. A total of 403 participants were given early surgery (n = 201) or delayed surgery (n = 202) treatmKentent randomly. There were no cases of giving up treatment observed throughout the research period. Furthermore, no statistically significant differences were discovered in terms of the baseline data between the two subgroups (Table 1). None of the patients were lost to follow-up throughout the present research. The eventual intention-to-treat analysis incorporated all of the patients (Fig. 2). The average time from injury to surgery was 1.4 days in the early surgery group and 4.6 days in the delayed group. The concluding appointment with the last randomly selected patient took place on Jan 31, 2022.

**Table 1:** Comparison of baseline data

	Early group (n = 201)	Delayed group (n = 202)	*p*
Age (Y, mean ± SD)	49.66 ± 3.5	50.28 ± 3.8	0.089
*Gender, no. (%)*			
Male	137 (68.16%)	143 (70.79%)	0.566
Female	64 (31.84%)	59 (29.21%)	
BMI (KG/cm^2^, mean ± SD)	22.98 ± 2.2	23.21 ± 2.4	0.317
Flail chest	114 (56.44)	109 (54.23)	0.578
Hemothorax	104 (51.74%)	101 (50.00%)	0.727
Pneumothorax	120 (59.70%)	115 (56.93%)	0.573
ISS, mean ± SD	14.91 ± 1.7	14.83 ± 1.5	0.617
Smoking History, no. (%)	71 (35.32%)	77 (38.12%)	0.561
Drinking History, no. (%)	85 (42.29%)	82 (40.59%)	0.730
*Living environment, no. (%)*			
Town	138 (68.66%)	142 (70.30%)	0.721
Countryside	63 (31.34%)	60 (29.70%)	
*Past medical history, no. (%)*			
Hypertension	75 (37.31%)	82 (40.59%)	0.500
Diabetes	59 (29.35%)	53 (26.24%)	0.485

### The primary endpoint and clinical efficacy

After follow-up, the length of stay in the intensive care unit (mean 4.63 days vs. 6.72 days, *p* < 0.001), the overall hospital stay (mean 10.15 days vs. 12.43 days, *p* < 0.001), and the number of ventilation days (mean 3.67 days vs. 4.55 days, *p* < 0.001) were significantly longer in the delayed surgery group than in the early surgery group. The hospitalization cost also decreased significantly in the early surgery group compared with the delayed group (mean 6900 USD vs. 7600 USD, *p* = 0.008). Concerning the surgical procedure time (mean 1.97 h vs. 2.01 h, *p* = 0.266), operative blood loss (mean 195.67 mL vs. 199.12 mL, *p* = 0.075), and 30-day all-cause mortality (2.49% vs. 3.47%, *p* = 0.485), there was no statistically significant difference between the two groups (Table 2).

**Table 2:** Comparison of primary end-point and clinical efficacy

	Early group (n = 201)	Delayed group (n = 202)	*p*
ICU days, day	4.63 ± 2.51	6.72 ± 3.24	< 0.001
LOS hospital days, day	10.15 ± 3.37	12.43 ± 3.59	< 0.001
Ventilation, n(%)	51 (25.37%)	64 (31.68%)	0.161
Ventilation days, day	3.67 ± 1.21	4.55 ± 1.68	< 0.001
Hospitalization cost, USD*10^3^	6.9 ± 2.7	7.6 ± 2.3	0.008
Surgical procedure time, hours	1.97 ± 0.35	2.01 ± 0.37	0.266
Operative blood loss, mL	195.67 ± 18.27	199.12 ± 20.48	0.075
30-days all-cause mortality	5 (2.49%)	7 (3.47%)	0.564

*ICU* Intensive care unit; *LOS* Length of stay

### The secondary endpoints

Before rib fracture fixation treatment, there was no significant difference in the inflammatory cytokine levels or serum infection marker levels between the two groups (*p* > 0.05). After rib fracture fixation treatment, the inflammatory cytokine levels and infection marker levels improved significantly in the early rib fracture fixation group compared with the delayed rib fracture fixation group (*p* < 0.05, Table 3).

**Table 3:** Comparison of the secondary end-points

	Before treatment			After treatment		
	Early group (n = 201)	Delayed group (n = 202)	*p*	Early group (n = 201)	Delayed group (n = 202)	*p*
*Inflammatory cytokine*						
TNF-α (pg/mL)	81.25 ± 18.47	80.39 ± 19.16	0.647	31.24 ± 12.10	37.26 ± 13.51	< 0.001
IL-6 (pg/mL)	125.92 ± 20.55	124.75 ± 19.89	0.562	59.76 ± 13.24	65.18 ± 15.84	< 0.001
*Infection index levels*						
CRP (mg/L)	30.15 ± 4.37	31.49 ± 4.42	0.613	5.06 ± 1.13	6.15 ± 1.07	< 0.001
PCT (ng/L)	0.46 ± 0.09	0.45 ± 0.08	0.095	0.24 ± 0.07	0.31 ± 0.11	0.135
WBC (× 10^9^/L)	18.05 ± 9.11	17.56 ± 8.97	0.716	12.74 ± 6.41	15.19 ± 7.29	0.017

### Complication evaluation

The most prevalent postoperative complications of rib fracture fixation include surgical site infection, pneumonia, and atelectasis. We found that 5 (2.49%) patients experienced surgical site infections in the early rib fracture fixation group, while there were 13 (6.44%) patients with surgical site infections in the delayed rib fracture fixation group, and there was no significant difference between the two groups (*p* = 0.055, Table 4). All surgical site infection patients were cured after antibiotic therapy, and the titanium rib fracture fixation system was not removed. The incidence of pneumonia (7.43% vs. 2.99%, *p* = 0.045, Table 4) and atelectasis (8.42% vs. 1.49%, *p* = 0.001, Table 4) was significantly higher in the delayed rib fracture fixation group than in the early rib fracture fixation group.

**Table 4:** Comparison of postoperative complications evaluation

	Early group (n = 201)	Delayed group (n = 202)	*p*
Surgical site infection	5 (2.49%)	13 (6.44%)	0.055
Pulmonary infection	6 (2.99%)	15 (7.43%)	0.045
Atelectasis	3 (1.49%)	17 (8.42%)	0.001

## Discussion

According to the findings of the current investigation, early rib fracture fixation may greatly decrease the length of stay in the intensive care unit, overall hospital stays, and ventilation days, and it can also reduce the hospitalization cost in MRF patients. The timing of rib fracture fixation does not influence the surgical procedure time, operative blood loss, 30-day all-cause mortality, or surgical site infection. Additionally, we also found that early rib fracture fixation can protect against hyperinflammation and improve infection. Moreover, it can also decrease the incidence of pneumonia and atelectasis after surgery. Iqbal [18] reported a similar result in their retrospective study that enrolled 102 patients. Early rib fracture fixation can decrease the length of stay with a better prognosis and fewer postoperative complications.

Rib fracture fixation treatment Multiple rib fractures have been used for hundreds of years to rebuild the chest wall and restore proper breathing mechanics, as well as to significantly reduce pain. However, recent clinical studies and meta-analyses have provided evidence of a potentially positive effect on mortality, ICU length of stay, and total hospital length of stay and a better outcome for patients with rib fracture fixation compared to a nonoperative treatment [12, 13, 15, 16, 20–22]. However, almost all studies and all the analyzed data sets were retrospective and were not specifically designed, and low levels of evidence in the literature limit the ability to make clear statements favoring one of the two treatment strategies by meta-analysis.

The timing of rib fracture fixation surgery is also controversial. One of the major reasons why only a limited number of studies have been conducted on the time point of surgery is that most studies focus on operative versus nonoperative treatment. Majak [23] demonstrated that rib fracture fixation surgery within 72 h may lead to better outcomes. Otaka [24] reported that rib fracture fixation surgery within 3 days after admission was associated with a shorter duration of mechanical ventilation and a shorter hospital stay through the Japanese diagnosis procedure combination database. Pieracci [25] analyzed a study that enrolled five hundred fifty-one patients and showed that surgical stabilization of rib fractures within one day was associated with favorable outcomes and less operative time. A review article reported that according to current research, rib fractures should be surgically stabilized as soon as possible, ideally within 72 h of injury [26]. Another recent meta-analysis including nine studies indicated that the optimal time of rib fracture fixation surgery in MRF patients was early (≤ 48–72 h after admission) and was associated with better clinical outcomes than delayed rib fracture fixation surgery (> 72 h after admission). Early fracture fixation surgery may improve pain and ventilatory function, as well as prevent complications resulting from chest trauma. In the present study, we found that early rib fracture fixation surgery in MRF patients (≤ 48 h after admission) can decrease the length of stay in the intensive care unit, overall hospital stay, and ventilation days. The timing of rib fracture fixation surgery did not affect the difficulty of the operation, and the surgical procedure time and operative blood loss were similar between the two groups. Hence, we also recommend that rib fracture fixation surgery should be completed as early as possible.

In the present study, we also evaluated inflammatory cytokine levels and serum infection marker levels. We also found that early rib fracture fixation surgery can decrease the inflammatory response and protect against infection. The probable reason may be that early completion of rib fracture fixation improved pain and other stress responses. Additionally, we also evaluated postoperative complications, such as surgical site infection, pneumonia, and atelectasis. There was no significant difference between the two groups in terms of surgical site infection, with few potential advantages in the early rib fracture fixation surgery group. The incidence of pneumonia and atelectasis decreased significantly in the early rib fracture fixation surgery group. These data were similar to the results of a previous retrospective study [18]. Of course, we cannot emphasize early surgery; early rib fracture fixation surgery was not recommended if the patient was unstable on admission or combined with other severe injuries (such as traumatic shock, severe traumatic brain injury, or multiple organ dysfunction syndromes).

The following were some of the study's limitations: Patients were not called back for study visits for a full evaluation of their quality of life in the context of activities of daily living (ADL); The morphology of the rib fractures, especially the amount of dislocation, was crucial for indicating rib fracture fixation, which was one of the major limitations of this study; The present research should have examined clinical parameters, including pain and subjective sleep quality.

## Conclusion

The findings of the present research suggest that early rib fracture fixation treatment may help to greatly minimize the decrease in the length of stay in the ICU, overall hospital stays, and ventilation days, decrease the inflammatory response and protect against infection, and greatly minimize the risk of postoperative complications following multiple rib fractures. The timing of rib fracture fixation does not influence the surgical procedure time, operative blood loss, 30-day all-cause mortality, or surgical site infection. Early rib fracture fixation (≤ 48 h) is a safe, rational, effective and economical strategy and worth clinical promotion. We look forward to more evidence-based medical research in the future as the present study's limitations.

## Data Availability

The datasets used and/or analyzed during the current study are available from the corresponding authors upon reasonable request.
